# Bioadhesive behaviors of HPMC E5: comparative analysis of various techniques, histological and human radiological evidence

**DOI:** 10.1038/s41598-024-52144-x

**Published:** 2024-01-22

**Authors:** Omar Y. Mady, Omar Dewedar, Noorelhoda Abdine, Hossam Zaytoon, Yusuf Haggag

**Affiliations:** 1 Department of Pharmaceutical Technology, Faculty of Pharmacy, Tanta University, Tanta, Egypt; 2 Department of Histology, Faculty of Medicine, Tanta University, Tanta, Egypt; 3 Department of Radiology, Faculty of Medicine, Tanta University, Tanta, Egypt

**Keywords:** Biomaterials, Biological models, Imaging, Drug delivery, Drug screening, Pharmaceutics, Target identification

## Abstract

Enhancing drug residence duration within the stomach offers distinct advantages for both localized and systemic effects. Numerous strategies have been proposed to extend drug residence time, with mucoadhesive polymers being a notable avenue. In this context, hydroxypropyl methylcellulose E5 has been employed as both a binding agent for granulating contrast metal powder and a mucoadhesive polymer, spanning various concentrations. The in vitro bioadhesion strength of the formulated tablets was gauged against the stomach lining of rabbits, for the quantification of bioadhesive forces. The temporal aspect of bioadhesion was evaluated through two approaches: one centered on gastric fluid dynamics and another proffered by the researchers, focusing on gastric wall kinetics. The results divulged a decline in bioadhesion force concomitant with high polymer concentrations. Histological examination of stained stomach sections revealed mucosal perturbations within the rabbit stomach. These disruptions exhibited an escalating trend in conjunction with elevated polymer concentrations, culminating in extensive disturbance at a 7.5% polymer concentration. The outcomes unveiled a direct relationship between polymer concentration increments and extended contact time. Subsequent radiological tracking of contrast metal behavior within a mature human stomach indicated a residence time of 6 h due to the entrapment of displaced components at disparate locations.

## Introduction

Oral formulations have attained a prominent stature among the diverse array of dosage forms developed for human administration. Various factors within the gastrointestinal milieu, encompassing surface area, pH levels, commensal microbiota, gastrointestinal transit duration, and enzymatic activity, exert notable influence on drug absorption processes^1^.

Upon scrutinizing the limitations inherent to conventional oral drug delivery systems, there has emerged a burgeoning interest in the exploration of novel delivery approaches. A pivotal concern affiliated with traditional oral medications lies in rapid gastric evacuation, contributing to diminished bioavailability of numerous drug entities. This poses a particularly formidable challenge for drugs reliant on absorption in the gastric or proximal small intestinal regions, as well as those grappling with absorption hindrances in the distal intestinal tract^2^. Additionally, drugs displaying low solubility within the alkaline pH milieu of the intestine can gain considerable solubility enhancement through sustained gastric retention^3^. Furthermore, select drugs such as captopril, metronidazole, and ranitidine HCl are susceptible to degradation within the colonic domain^4^.

In instances where therapeutic efficacy necessitates frequent dosing due to short drug half-lives, the conceptualization of an oral sustained-controlled release formulation endowed with gastric retention attributes emerges as a potential solution. By facilitating gradual drug release within the stomach, this formulation not only counteracts the aforementioned limitations but also sustains optimal systemic drug concentrations over extended temporal spans^5^.

Gastro-Retentive Drug Delivery Systems (GRDDS), known as RegenerateGastro-Retentive Drug Delivery Systems, have demonstrated notable effectiveness in treating localized afflictions such as gastric and duodenal ulcers, as well as esophagitis. The mechanism underlying this efficacy lies in the targeted elimination of the entrenched Helicobacter pylori bacteria residing within the submucosal tissue of the stomach. Beyond their systemic impact, the distinct attribute of GRDDS resides in their ability to exert localized therapeutic influence, particularly in managing these conditions^6,7^.

Researchers have harnessed diverse methodologies to extend gastric residence duration and sustain controlled drug release within Gastro-Retentive Drug Delivery Systems (GRDDS). The developmental challenges of crafting efficacious GRDDS encompass transcending the impediments associated with the stomach's rapid gastric emptying rate, while concurrently maintaining an appropriate rate of drug release that endures for an extended interval prior to metabolic degradation within the body^5^.

Several techniques have been employed to augment gastric residency and enable sustained drug release within Gastro-Retentive Drug Delivery Systems (GRDDS). These encompass the utilization of formulations with heightened density, polymer swelling, magnetic force manipulation, hybrid approaches involving polymer swelling and effervescence, and the principle of mucoadhesion^5^.

Mucoadhesive drug delivery systems exhibit the capability to heighten drug concentration within specific locales through intimate adherence to the absorption site. Moreover, this sustained and close interaction contributes to the enhancement of drug permeability, extending to peptides and proteins. In contemporary times, such delivery systems have been conceived for diverse administration routes, spanning buccal, sublingual, nasal, rectal, and vaginal routes, serving both systemic and localized therapeutic agendas^8^.

The efficacy of mucoadhesion critically hinges on the capacity of the mucoadhesive polymeric material to sustain adhesion to the mucosal membrane. Consequently, the judicious selection of the polymer material utilized in the formulation, coupled with the biochemical attributes of mucosal membranes across various bodily regions, assumes pivotal roles in dictating mucoadhesive performance^9^.

The polymeric composition employed in the formulation of pharmaceutical dosage forms stands as a pivotal determinant for achieving optimal mucoadhesion. An exemplary mucoadhesive material should swiftly establish adhesion to the designated mucosal area, facilitate unhindered drug release, exhibit biocompatible and biodegradable characteristics, safeguard the drug from enzymatic degradation, and augment drug penetration capabilities^10^.

Hydroxypropyl Methylcellulose (HPMC), a semisynthetic cellulose derivative, has emerged as a cornerstone pharmaceutical excipient across diverse pharmaceutical technologies. Within the realm of mucoadhesive drug delivery systems, HPMC plays a crucial role, finding application across numerous therapeutic domains. Its inherent hydrophilic and advantageous attributes render it an ideal polymer for drug delivery^11^. Functioning as a non-ionic polymer, HPMC bestows a range of advantages to drug delivery systems, encompassing minimal susceptibility to drug interactions, consistent drug release profiles, and pH-independent behavior^12,13^. Furthermore, the swelling and wetting attributes of HPMC constitute pivotal determinants influencing both the mucoadhesive potency and the duration of interaction between the polymer and mucosal surface.

The mucoadhesive mechanism of HPMC polymer entails the formation of hydrogen bonds via hydroxyl groups, coupled with the intertwining of polymer chains with mucins. As a mucoadhesive excipient, HPMC can be judiciously applied to the mucosal linings of the oral cavity and the gastrointestinal tract, effectively harnessing its pronounced mucoadhesive attributes^8,14,15^.

Consequently, the fundamental aim of this research endeavors to comprehensively investigate the bioadhesive attributes inherent to hydroxypropyl methyl cellulose E5 (HPMC E5) through in-vitro assessments. This analysis entails the judicious utilization of a spectrum of both established and innovative techniques, with the polymer serving as a binding agent for a tablet infused with a contrast metal. The investigation further extends to the evaluation of the polymer's influence on the rabbit stomach, encompassing a histological examination to elucidate the bioadhesive vigor and temporal dynamics of polymer-mucosa interaction within this biological context. Subsequently, the study deeply investigates the behavior of the contrast metal powder, granulated in tandem with the polymer and subsequently compressed into tablet form. The ensuing radiological observations provide substantiating evidence for the behavior of this contrast-laden formulation within the human stomach.

## Materials and methods

### Materials

Hydroxypropyl methylcellulose (HPMC) was procured in the form of generous contributions from Sigma Pharmaceutical Company, located in Quesna, Egypt. Barium sulfate, an essential component, was sourced from Alfa chemical group located in 6th of October, Egypt. Hydrochloric acid and periodic acid, were acquired from El Gomhoria for chemical trade in Egypt. Schiff's reagent, along with xylol and Canda balsam, pivotal elements for the experimental procedures, were obtained from the reputable supplier Sigma-Aldrich based in Missouri, USA.

### Contrast metal granulation

Table 1 outlines the comprehensive composition of the diverse formulations subjected to experimentation. The stipulated quantities of barium sulfate and hydroxypropyl methylcellulose (HPMC E5) powders were meticulously measured and subsequently amalgamated within a polyethylene bag for a duration of 2 min. The ensuing amalgamation was attained by introducing a calculated volume of 85% hydroalcoholic solution (1.25 ml) to a 10 g amalgamation of powder via a mortar and pestle, effectively yielding a cohesive mass. These moist granules were then precisely generated by subjecting the mass to a 1 mm sieve (impact laboratory test sieve, UK). Subsequently, the freshly formed granules were subjected to a drying process in an oven set at 60 °C for a duration of 25 min. Following this drying phase, the granules underwent an additional sieving procedure using a 1 mm sieve. The desiccated granules were stored within a desiccator, reserved for subsequent in-depth analysis.

**Table 1:** Composition of various barium sulfate granule formulations.

Composition	F1	F2	F3	F4	F5	F6	F7
Barium sulfate	10 g	10 g	10 g	10 g	10 g	10 g	10 g
HPMC (E5)	0.05 g	0.1 g	0.15 g	0.2 g	0.25 g	0.5 g	0.75 g
Hydro-alcohol	1.25 ml	1.25 ml	1.25 ml	1.25 ml	1.25 ml	1.25 ml	1.25 ml

### Tablet pressing

The prepared granules were precisely compacted into tablets using a multi-punches compression machine, specifically the Stokes rotary compression machine (model: 912-5121) originating from the USA. The employed machine was equipped with round flat punches featuring a diameter of 14 mm. Each tablet was manually created by applying a consistent quantity of the prepared granules (1.4 g) and subjecting them to compression under a uniform pressure.

### In vitro bioadhesion strength

In order to assess the bioadhesive potency of the formulated contrast metal tablets, the mucosal membrane of the rabbit stomach was employed as a biological model^16^. The experimentation utilized the stomach of a male albino rabbit, weighing 2 kg and sourced from the Tanta animal house. All procedures involving animal subjects were ethically approved and rigorously overseen by the Animal Ethics Committee of the Faculty of Pharmacy at Tanta University, Egypt (Ethics code: TP/RE/2/23M-0012) and all experiments were performed by the guidelines and regulations of this committee. Adherence to the ARRIVE guidelines 2020 was stringently observed, ensuring optimal animal welfare and minimizing potential discomfort. The animal was anesthetized using ketamine HCl before the stomach was removed for experimentation.

Following removal, the rabbit's stomach was carefully cleansed using a saline solution and then preserved in a Tyrode solution. A longitudinal incision facilitated the extraction of the mucosal surface from the stomach. The obtained mucosal fragments were sliced into pieces exceeding the surface area of the tablet's flat side. These fragments were then firmly affixed to a slide with the mucosal surface positioned upwards, securely adhered using cyanoacrylate glue. Prior to contact, the rabbit stomach's surface was moistened with 0.1N HCl (2 drops). Subsequently, the tablets were tied to a thread and brought into contact with the rabbit's stomach.

On the opposite side of the tablet, a consistent weight (100 g) was applied for a duration of five minutes (referred to as the preload time).

One end of the thread was attached to an iron stand balance, while the slide was fixed onto the ground. The other side of the balance was connected to a small polypropylene bag that had been pre-weighed. A burette was employed to supply water into the polypropylene bag at a constant rate until the tablet detached from the stomach tissue. The quantity of water accumulated in the bag was recorded and expressed as the weight in grams necessary for the tablet to detach^17,18^. The following equations were applied to calculate the force of adhesion and bond strength^18,19^:

1 $${\text{Force}}\;{\text{of}}\;{\text{Adhesion}}\;\left( {\text{N}} \right) = \left[ {{\text{Bioadhesive}}\;{\text{Strength}}\;\left( {\text{g}} \right) \times {9}.{81}} \right]/{1}000$$

2 $${\text{Bond}}\;{\text{Strength}}\;\left( {{\text{Nm}}^{2} } \right) = {\text{Force}}\;{\text{of}}\;{\text{Adhesion}}/{\text{Tablet}}\;{\text{Surface}}\;{\text{Area}}$$

The reported results represent the average values obtained from six tablets.

### Determination of in-vitro bioadhesion time using different methods

#### USP disintegration apparatus (method 1—direct assessment)

The evaluation of the in-vitro residence time of the prepared tablets was carried out using the USP disintegration apparatus, specifically designed to determine the time required for these tablets to detach from a composite assembly comprising a fixed movable unit (tablet-rabbit stomach mucosa-plastic slab)^20^. The rabbit stomach was meticulously sectioned into circular pieces, which were then affixed to a round plastic slab with the mucosal side oriented upward, using cyanoacrylate glue. The mucosal surface of the rabbit was moistened with 0.1N HCl (2 drops), following which the contrast metal tablet was positioned on the mucosa. A consistent weight of (100 g) was subsequently applied over the tablet for a duration of five minutes, denoted as the preload time. The assembled plastic slab, bearing the stomach mucosa and the affixed tablet, was inserted into a tablet disintegration machine, specifically the Copley disintegration tester (model: DTG 2000) from the UK^21^. The disintegration vessel was charged with a solution of 0.1N HCl at a temperature of 37 ± 0.5 °C, simulating the gastric fluid environment.

The plastic slab was vertically situated within the apparatus, allowed to oscillate up and down, thereby causing the tablet to be fully immersed in the solution at its lowest point and to surface at its highest point. The time requisitioned for the comprehensive erosion or detachment of the tablet from the mucosal surface was meticulously recorded as the mean value derived from triplicate determinations.

#### USP disintegration apparatus with regard to gastric motility (method 2—motility-linked assessment)

The in-vitro residence time of the formulated contrast metal tablets was evaluated through the measurement of the duration needed for detachment from the rabbit stomach mucosa, utilizing the USP disintegration apparatus. In this approach, the rabbit stomach was methodically sectioned into circular fragments, and these were firmly attached to round plastic slabs, ensuring the mucosal side was oriented upwards, and utilizing cyanoacrylate glue. The circular plastic slab, bearing the stomach piece, was securely positioned within the sieves of the disintegration apparatus tubes, also ensuring the mucosal side faced upwards. The composite assembly of the round plastic slab housing the stomach fragment possessed a surface area smaller than that of the disintegration apparatus tube.

The formulated tablet was carefully placed within a tube of the disintegration apparatus, allowing it to naturally adhere to the surface of the stomach mucosa affixed to the plastic slab, without the need for any additional fixation weight. This configured assembly was then permitted to oscillate vertically, thereby immersing the tablet completely in the solution at the lowest point and elevating it to the surface at the highest point. The time required for complete erosion or detachment of the tablet from the mucosal surface was accurately recorded as the mean value derived from triplicate determinations.

### Histological examination of stomach segments

Upon the conclusion of the bioadhesion evaluations, specific segments of the rabbit gastric mucosa were subjected to a histological assessment, involving staining with the periodic acid-schiff (PAS) stain, a method aimed at highlighting neutral mucopolysaccharides as outlined^22^.

#### Procedure for periodic acid-Schiff staining

The sections of rabbit gastric mucosa underwent a sequence of preparatory steps. First, the sections were deparaffinized and subsequently subjected to progressive hydration via escalating alcohol concentrations for a span of 2 min for each respective grade. Thereafter, the sections were immersed in a 1% solution of periodic acid for a duration of 8 min, followed by rinsing under a stream of running tap water for a minute. Following this, the sections were introduced into Schiff's reagent for a period of 15 min until a light pink hue was attained. Subsequently, the sections were rinsed under running tap water for 5 min until they assumed a dark pink color. The ensuing sections were subsequently dehydrated through an ascending series of alcohol grades, clarified with xylol, and ultimately mounted in Canada balsam. This comprehensive staining protocol culminated in the development of a deep red or magenta coloration, indicative of the presence of stained mucopolysaccharides within the sections.

#### Morphometric analysis of PAS stain’s optical density

The software program Image J, version 1.46, was harnessed for the examination of sections from the fundic mucosa across all experimental groups. The optical density of the PAS stain was quantified across 5 distinct random microscopic fields for each individual specimen, with a magnification power set at ×200. The obtained data were subjected to rigorous statistical analysis employing SPSS software, version 13 (SPSS Inc., Chicago, IL, USA). This encompassed one-way analysis of variance (ANOVA) followed by Tukey’s test for the comparison of diverse groups against the control group. The outcomes were expressed in the format of mean values accompanied by their standard deviations (SD). Significance levels were determined based on probability values (*P*), with a value of *P* < 0.05 signifying statistical significance, *P* < 0.001 denoting high significance, and *P* > 0.05 indicating non-significance, in accordance with established practices^23^.

### In vivo radiological imaging of the tablet

The radiographic evaluation of the tablet was carried out through fluoroscopy on human subjects of mature age, following the stipulated ethical protocol sanctioned by the Tanta College of medicine ethical committee (Ethical code: 36264MS310/9/23) and all experiments were performed by the guidelines and regulations of this committee. The radiographic procedure was conducted within the fluoroscopy unit situated in the Radiology Department of the Tanta University Educational Hospital.

#### Volunteer preparation

A cohort of three healthy male volunteers, ranging in age from 27 to 30 years and displaying body weights within the interval of 70–95 kg, participated in the study after undergoing a comprehensive informed consent process. The volunteers were instructed to observe an overnight fast before the experiment, and each individual ingested a single tablet from formulations F5, F6, or F7 (as indicated in Table1), paired with 100 mL of water. The fasting state was upheld throughout the entire course of the experiment.

#### Fluoroscopy procedure

In preparation for the radiological examination, participants were directed to eliminate any metallic objects or clothing that could potentially obstruct the targeted body area under examination. Subsequently, they were suitably positioned in an upright posture on the fluoroscopy unit table. Employing a digital fluoroscopy system (Flexavision FD, Shimadzu, Japan), the fluoroscopic images were captured using a low-dose fluoroscopic cine technique, characterized by a low tube potential (80 kVp) and adjustable milliampere seconds (mAs), with the system automatically adapting the mAs based on individual body morphology.

Sequential images of the abdominal region were acquired at various intervals during the procedure, documenting both the tablet's position and configuration. This imaging schedule encompassed an immediate post-swallowing image, in addition to subsequent images captured every half an hour until the tablet traversed the anticipated gastric region. The fluoroscopy methodology involves gauging the attenuation extent of the x-ray beam post-passage through the human body. The resultant x-ray photons emanating from the body are captured by a digital imaging receptor, which then projects the ensuing image onto a monitor following computational processing. The presence of barium sulfate within the tablet formulation bestows a heightened attenuation of the x-ray beam, rendering the tablet radiopaque, and consequently appearing as a white entity in the x-ray images. Conversely, other abdominal structures manifest as varying shades ranging from white to black with gradations of gray in between, reflective of the distinct degrees of x-ray attenuation within each anatomical region^24^.

## Results and discussion

The principal objective of this phase of the study was to devise a novel tablet dosage form incorporating a contrast metal, utilizing Hydroxypropyl Methylcellulose E5 (HPMC E5) as a pivotal binding agent. Barium sulfate was specifically chosen as the contrast metal due to its ability to be visualized in gastrointestinal transit via X-ray imaging. Its non-compressible nature eliminated concerns related to tablet disintegration, while its presence within the disintegrated granules enabled tracking post-disintegration. The wet granulation technique was adopted to facilitate the effective compression of contrast metal granules into tablets, ensuring optimal dispersal of HPMC polymer within barium sulfate, given their fine powder nature. This approach served a dual purpose: first, enhancing the compressibility of barium sulfate for tablet formation; second, capitalizing on HPMC's recognized mucoadhesive properties^8,14,15^.

### Wet granulation technique

The application of wet granulation was chosen to achieve both adequate compressibility of contrast metal granules and thorough incorporation of HPMC polymer with barium sulfate, due to their fine powder characteristics. Wet granulation, a well-established approach in pharmaceutical technology, entails blending drug substances with excipients while introducing a binder liquid to form granules. Following drying, sieving, and subsequent compression, tablets are obtained. This method enhances the compressibility of granules compared to powders, yielding tablets with improved hardness and reduced powder content. The optimal amount of binding solution was determined as 1.25 ml. Variations in the volume of binding solution were found to either result in granules with higher powder content or a softer mass, both undesirable outcomes. Maintaining the constant binding solution volume across different formulations (as outlined in Table 1) ensured consistent granule quality.

### Tablet pressing and adhesive properties

Tablet pressing involved transferring the prepared granules onto punches. The extent of tablet picking increased with higher concentrations of the bioadhesive polymer (HPMC). This phenomenon might be attributed to the hygroscopic nature of HPMC. Notably, the act of holding pressed tablets between fingers resulted in the deposition of a white film on the fingers, with this effect diminishing as HPMC concentration increased. This observation hints at the possibility that the quantity of binder might be insufficient for optimal binding of the contrast metal (barium sulfate).

### In-vitro bioadhesion strength assessment

**Figure 1:**
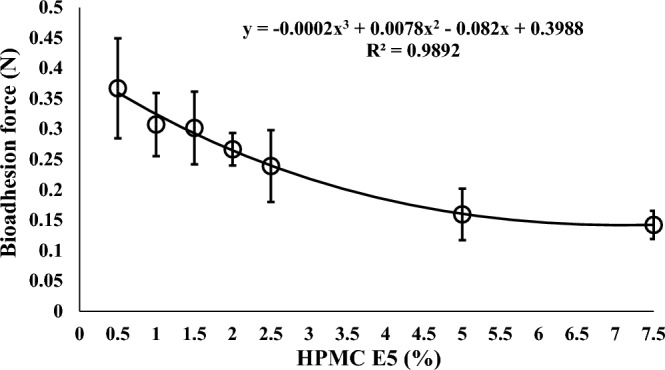
The adhesion force of contrast metal tablets prepared with varying concentrations of the bioadhesive polymer.

The investigation involved simulating the bioadhesion strength of the tablet surface to the stomach membrane in situ, employing a methodology detailed in^17–19^. The resultant findings are graphically presented in Fig. 1.

The analysis of the presented figure unmistakably reveals an intriguing pattern: as the concentration of the bioadhesive polymer (HPMC) escalates, the bioadhesive force of the prepared tablets experiences a noticeable reduction. This relationship conforms to a polynomial model, with a regression coefficient of (R^2^ = 0.9892), highlighting a robust correlation between the bioadhesive forces and the concentrations of the polymer. However, these results appear to defy the expected concept of adhesive force between surfaces, where heightened adhesive polymer concentration typically corresponds to increased adhesion forces between materials.

This paradoxical outcome can potentially be ascribed to the intricate interplay of numerous factors intrinsic to the in-situ simulation approach. The utilization of a biological membrane, specifically the stomach surface of a rabbit, introduces a degree of biological complexity that seeks to mirror the conditions found within humans. Moreover, the moistening of the stomach surface with 0.1 N HCl adds another layer of complexity to this experimental framework.

A notable point of consideration stems from prior research indicating that elevating the viscosity of Hydroxypropyl Methylcellulose (HPMC) could lead to reduced compact porosity, subsequently affecting surface roughness^25^. Surface roughness is a pivotal factor that exerts influence on the contact angle, a measure of a solid surface's wettability^26^. The Wenzel model posits that augmented surface roughness enhances wettability by facilitating better contact between the liquid and the solid surface^27^. Increased contact angle values signify reduced surface wettability, potentially contributing to diminished adhesive strength^28^. The enhanced viscosity of HPMC may contribute to a smoother and more hydrophobic surface of HPMC compacts, potentially leading to reduced adhesive strength.

These conclusions, drawn from laboratory experiments, have guided us toward a more comprehensive examination of the rabbit stomach segment surfaces subsequent to conducting the bioadhesion tests. Such an investigation aims to ascertain whether any adverse effects resulting from the contrast metal or the bioadhesive polymer might contribute to the observed phenomenon.

### Histological evidence of the rabbit stomach segments surface

Staining serves as a pivotal technique in histology, serving both to accentuate significant tissue features and augment tissue contrast for improved microscopic visualization. This technique finds utility in enhancing cell visibility under a microscope and can also facilitate differentiation between live and deceased cells within the tissue. A well-established stain in the realm of pathology is the Periodic-Acid Schiff (PAS) stain, renowned for its diagnostic application. PAS stain aids in the identification of structures rich in carbohydrate macromolecules, such as glycogen, glycoproteins, and proteoglycans. These macromolecules are commonly found in connective tissue, mucus, and basal laminae.

**Figure 2:**
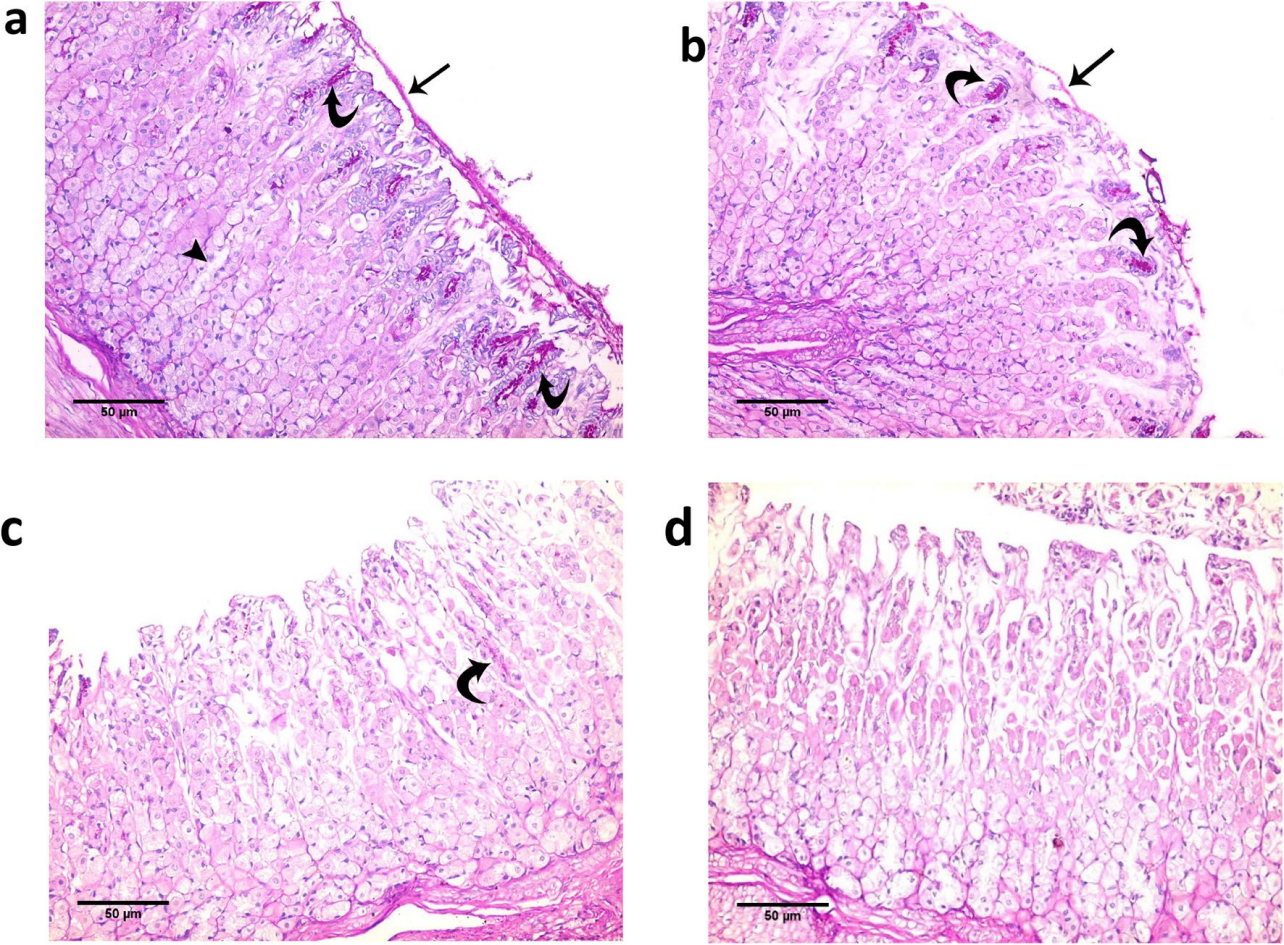
The photomicrograph of the fundic gastric mucosa pf the rabbit segments: (**a**) Control group (**b**) After using 1% HPMC (**c**) After using 2.5% HPMC (**d**) After using 7.5% HPMC. NB: (PAS × 200).

The photomicrographs in Fig. 2 provide visual insight into the histological characteristics of the fundic gastric mucosa. In the control group (Fig. 2a), distinct features become evident. A notably strong PAS-positive material (indicated by an arrow) envelops the surface of the gastric mucosa. This material is also present in the pits and isthmus of the gastric glands (as depicted by curved arrows). Conversely, no discernible PAS-positive reaction is observed at the base of the glands (arrowhead).

In comparison, the photomicrograph of the fundic gastric mucosa from the group studied to evaluate the bioadhesion force of tablets prepared with 1% HPMC (Fig. 2b) reveals a different pattern. A comparatively weaker PAS-positive reaction is evident in the surface mucous coat (arrow), while remnants of positive PAS reaction are still discernible at the isthmus of certain gastric glands (curved arrows). This outcome strongly suggests a disturbance in the surface mucosal layer of the studied segment.

Staining techniques, particularly the PAS stain, have offered valuable insights into the composition and structural characteristics of the gastric mucosa. The observed differences in PAS-positive reactions between the control group and the group exposed to bioadhesive tablets underscore the potential impact of the tablet formulation on the mucosal layer. Further examination and interpretation are warranted to ascertain the broader implications of these findings within the context of the study's objectives.

Further insights emerge from the photomicrographs, each corresponding to distinct concentrations of HPMC in tablet preparation, as they reveal the extent of impact on the gastric mucosal layer.

The photomicrograph from the group that investigated tablets formulated with 2.5% HPMC (Fig. 2c) presents a remarkable reduction in PAS reaction at the isthmus of the gastric glands. Additionally, no discernible reaction is evident at the surface of the glands. These findings collectively point to a heightened disruption within the mucosal layer of the segment under study.

For tablets prepared with 7.5% HPMC (Fig. 2d), the results are even more pronounced. The photomicrograph of the fundic gastric mucosa reveals a complete absence of any PAS reaction, both at the surface of the glands and the isthmus of the gastric glands. These observations consolidate the notion that the utilization of HPMC plays a decisive role in perturbing the mucosal layer of the rabbit stomach, with the degree of disturbance escalating in tandem with the concentration of the bioadhesive polymer. Furthermore, the application of 7.5% HPMC appears to erase the mucus film that typically overlays the mucosal layer.

**Figure 3:**
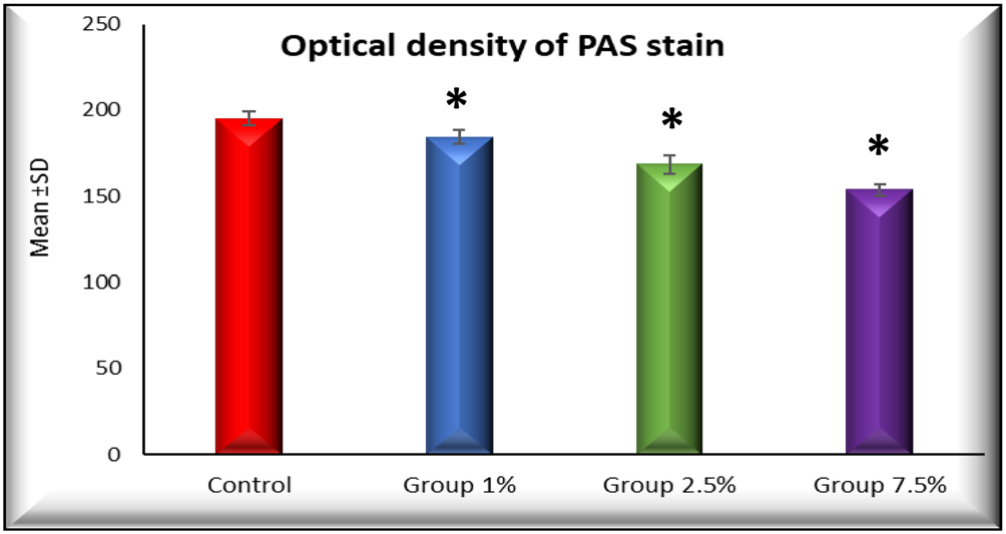
Histogram of the mean optical density of PAS in different studied groups of the fundic mucosa. * = Highly significant, *P* value =  < 0.001.

The application of Image J analysis software allowed for the quantification of PAS stain optical density, which proved to be significantly reduced across all experimental groups as compared to the control group. Comprehensive tabulation of these results can be found in Table 2 and Fig. 3, shedding light on the varying degrees of optical density alteration within the studied groups.

**Table 2:** The mean optical density of PAS in different studied groups.

Groups	Optical density of PAS stain			ANOVA	
	Range	Mean ± SD		F	*P* value
Control	190–200	195.200 ± 3.962		93.510	< 0.001*
Group 1%	180–189	184.400 ± 4.037			
Group 2.5%	161–175	168.400 ± 5.273			
Group 7.5%	149–158	153.600 ± 3.362			
TUKEY’S Test					
C&1%	C&2.5%	C&7.5%	1%&2.5%	1%&7.5%	2.5%&7.5%
0.005*	< 0.001*	< 0.001*	< 0.001*	< 0.001*	< 0.001*

* = Significant.

*SD* standard deviation.

The histological assessments reveal a clear link between the concentration of HPMC in tablet formulation and the ensuing perturbations within the gastric mucosal layer. These findings lay the foundation for understanding the dynamic interplay between bioadhesive polymers, mucosal integrity, and the outcomes of in-vitro experiments.

The rationale underpinning bioadhesion technology hinges on the establishment of physicochemical interactions between bioadhesive polymers and the mucosal membrane^8,29^, thereby extending the residence time of drug delivery systems within the targeted site, whether for local or systemic effects. This theoretical framework aligns with well-established literature on the subject, which attributes increased residence time to heightened bioadhesion forces. However, the experimental results presented in Fig. 1 challenge this conventional understanding by showcasing a reverse trend: increased bioadhesive polymer concentration leads to a decrease in bioadhesion force.

The underlying reasons for this counterintuitive phenomenon are elucidated by examining the histological study outcomes. Specifically, analysis of the rabbit stomach segments after bioadhesion tests reveals an escalating disturbance of the mucosal membrane as the bioadhesive polymer concentration increases. Consequently, this disturbance is proposed to be responsible for the observed reduction in bioadhesion force. It is evident that the polymer's interaction with the mucosa disrupts its structural integrity, thereby undermining the expected adhesive forces between the polymer and the mucosal membrane.

### In-vitro bioadhesion time measurement: a comprehensive approach

In the context of in-vitro bioadhesion time measurement, existing methods, while valuable, often do not encapsulate the full complexity of the physiological conditions. A prevailing method involves fixing mucosa to a slide and placing the film on its surface before immersing the assembly in a dissolution vessel^30^. This approach neglects the intricate interplay of stomach muscle contractions, which contribute significantly to the detachment of the film from the mucosa. Additionally, the omission of the stomach's own movement and mixing mechanisms introduces further limitations to the simulation.

A more comprehensive attempt at mimicking the stomach environment was introduced by Nafee and colleagues, who utilized a modified USP disintegration apparatus^21^. In this method, the mucosa and bioadhesive tablet assembly is vertically aligned and moves as a single unit within the apparatus. This accounts for both the peristaltic action of the stomach muscles and the interaction between the tablet and mucosa in a more holistic manner. This improved methodology, referred to as the "modified method," offers a more accurate representation of in-vivo conditions compared to traditional in-vitro techniques.

The intricate dynamics of bioadhesion, particularly in the context of varying polymer concentrations, reflect a complex interplay between physicochemical interactions, mucosal integrity, and the subsequent residence time of drug delivery systems. The experimental results underscore the importance of adopting multifaceted approaches that more faithfully emulate physiological conditions to yield a more comprehensive understanding of bioadhesive behavior.

#### Modified method for measuring the bioadhesion time

**Figure 4:**
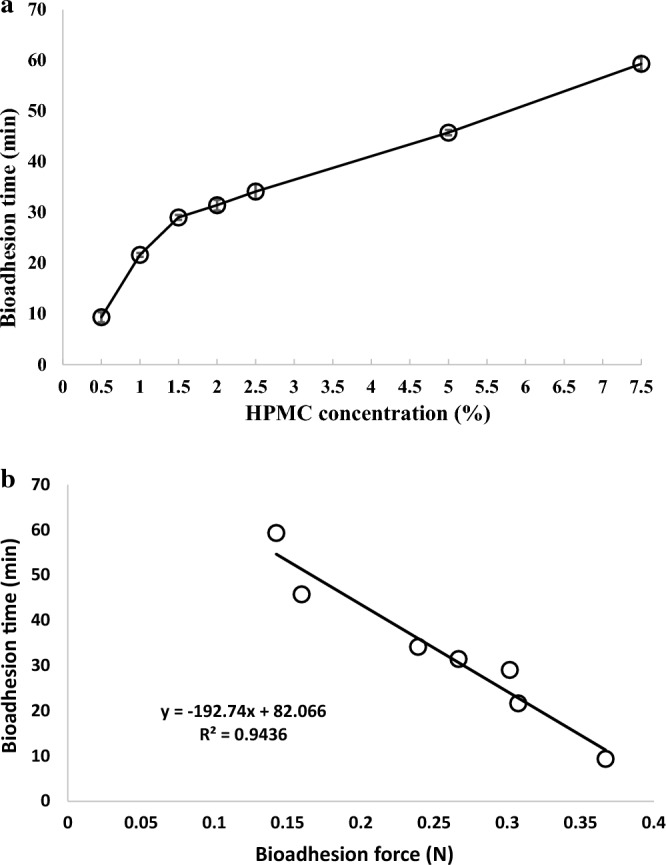
(**a**) Represent the correlation between the tablet bioadhesion time and the concentration of HPMC used. (**b**) Correlation between the bioadhesion time and bioadhesion force of different contrast metal tablets prepared by using different concentrations of bioadhesive polymer.

Upon a closer examination of the experimental data, it becomes evident that bioadhesion time and bioadhesion force, although interconnected, might be influenced by distinct underlying mechanisms. Figure 4a portrays the relationship between polymer concentration and bioadhesion time, revealing an intriguing trend: increasing polymer concentration results in prolonged bioadhesion time. This phenomenon is characterized by two distinct phases with varying rates of increase.

To decipher this seemingly contradictory behavior, an exploration of potential contributing factors is warranted. Notably, the non-ionic polymer HPMC exhibits limited water uptake and swelling, as reported by previous studies^21,31^. Mortazavi and Smart's research aligns with these findings, indicating that HPMC's prolonged adhesion duration is attributed to its modest mucoadhesive strength coupled with limited hydration^31^. The long-lasting adhesion, despite relatively weak mucoadhesive forces, corresponds well with the observations of this study^32,33^.

Intriguingly, HPMC also possesses hygroscopic properties and can be soluble at lower molecular weights. This introduces an alternative perspective to explain the observed increase in bioadhesion time with higher polymer concentrations. The proposed hypothesis revolves around HPMC's dual role as a glue-like substance and its ability to reduce fluid accumulation between the tablet surface and the mucosal membrane. When a contrast metal tablet containing HPMC contacts the mucosal membrane, dissolved polymer acts akin to glue, adhering the tablet to the tissue. This effect intensifies as polymer concentration increases, ultimately yielding longer contact times.

To substantiate this notion, the authors introduced concave surface tablets and performed a contact time test. The results indeed showcased extended contact times for concave tablets compared to flat ones. The concave tablets' configuration facilitates fluid diffusion between the tablet surface and the tissue, gradually dissolving the polymer and releasing the tablet. This experimental finding corroborates the notion that HPMC's adhesive nature, combined with its hygroscopic behavior and solubility, engenders prolonged contact times by forming a "glue" effect between the tablet and the mucosal membrane.

The observed inverse relationship between bioadhesion force and bioadhesion time (Fig. 4b) underscores the intricate interplay of various factors, including polymer concentration, hydration characteristics, and the adhesive and solubility properties of HPMC. The complexity of these interactions highlights the multifaceted nature of bioadhesion phenomena, further necessitating a comprehensive approach to elucidate the mechanisms at play.

#### Determination of the bioadhesion time with a simulation concerned gastric motility

**Figure 5:**
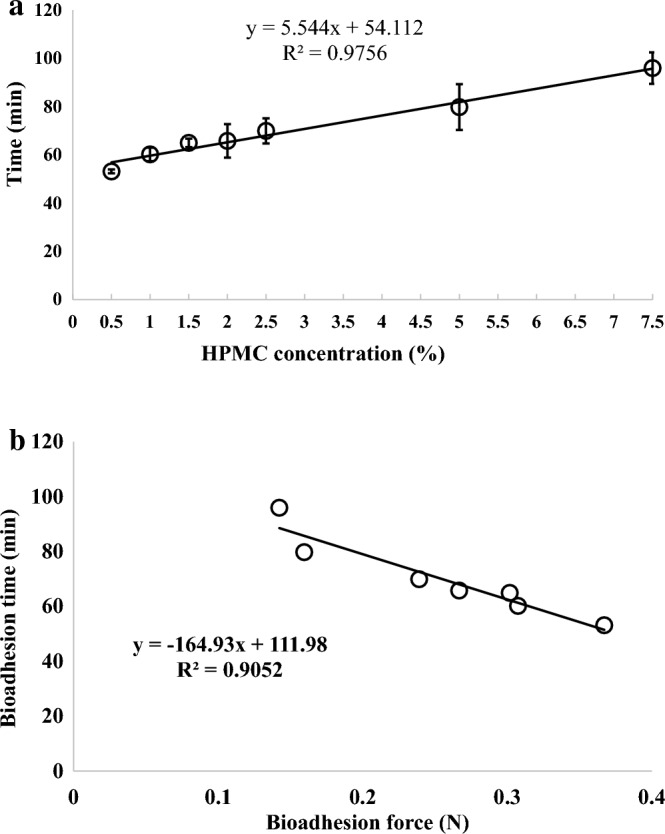
(**a**) Represent the correlation between the tablet bioadhesion time and the concentration of HPMC used. (**b**) Correlation between the bioadhesion time and bioadhesion force of different contrast metal tablets prepared by using different concentrations of bioadhesive polymer.

To investigate deeper into the interplay between polymer concentration, bioadhesion time, and bioadhesion force, the authors introduced an innovative simulation procedure in Fig. 5a. This new approach sought to more closely mimic the dynamic conditions within the stomach, further enhancing the validity of the study’s outcomes.

In this refined simulation, plastic plates were strategically fixed within sieves, a design that prevented tablets and stomach mucosa from flipping down during the procedure. This modification aimed to more accurately represent the actual conditions within the stomach and provided a closer approximation of the physiological processes involved. The complex interactions were symbolized as follows: the movement of the disintegration tube mirror the movement of gastric contents, while the assembly’s up-and-down movement within the tube represented stomach contraction.

The results were telling. Figure 5a reveals a clear correlation between polymer concentration and bioadhesion time. As polymer concentration increased, bioadhesion time followed suit. The controlled and consistent nature of this simulation procedure likely contributed to the direct relationship observed between the polymer concentration and bioadhesion time.

Moreover, Fig. 5b underscores the inverse proportion between bioadhesion time and bioadhesion force, as discussed earlier. This reinforced the intricate balance between these two parameters and showcased how subtle variations in experimental conditions could yield profound insights into bioadhesion behavior.

### In-vivo evaluation of bioadhesive tablet (X-ray studies)

Radiography served as a valuable tool to study the behavior of bioadhesive tablets as they traversed the human stomach. This non-invasive imaging technique allowed for the real-time tracking of contrast metal tablets (F5, F6, or F7), which were formulated with varying concentrations (2.5, 5, and 7.5%) of the bioadhesion polymer. The selection of these concentrations was strategically based on the bioadhesion time capabilities of the prepared tablets, ensuring that the experiment could be effectively carried out.

**Figure 6:**
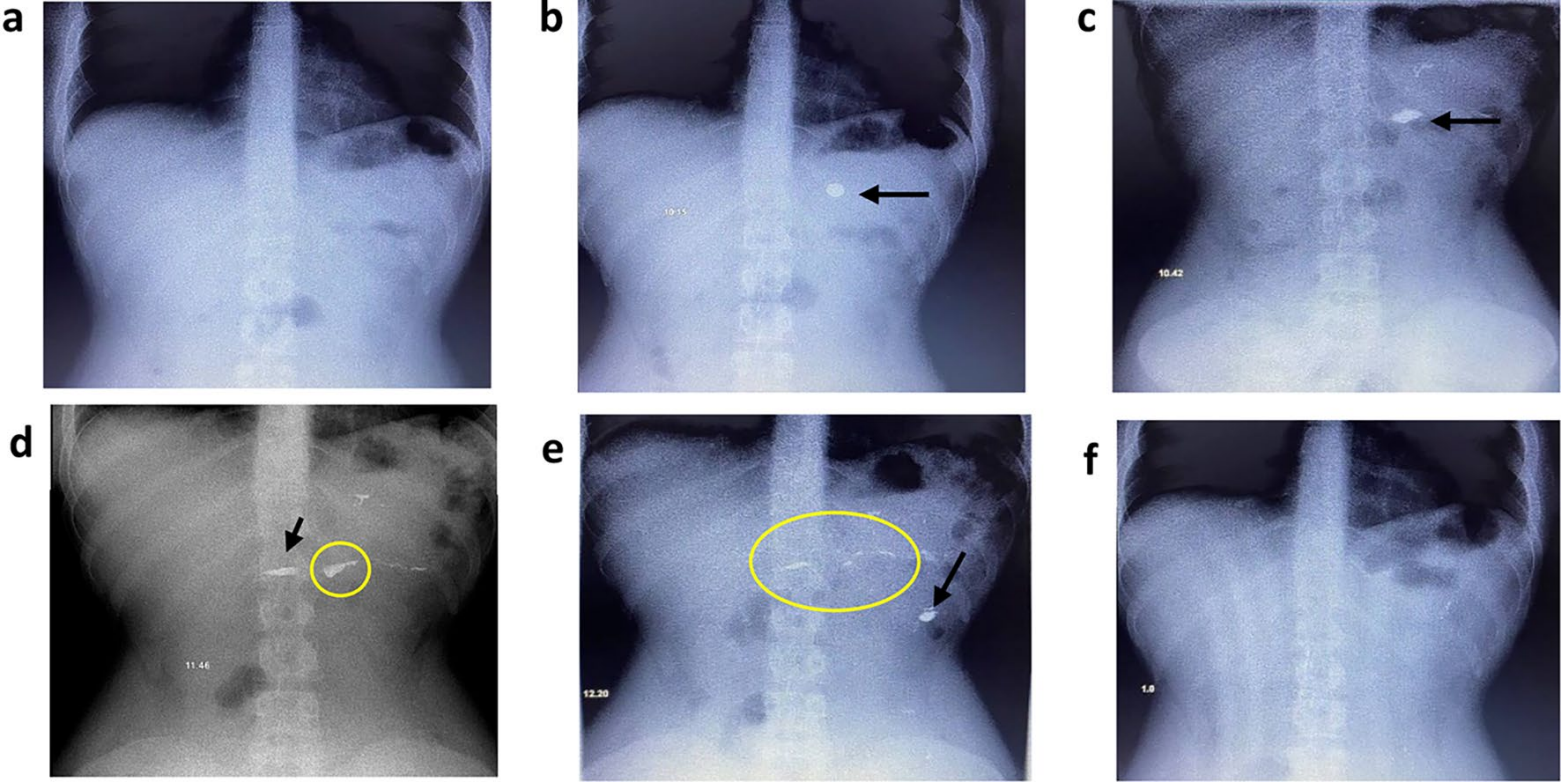
Radiographic images of the location of the contrast metal tablet (F5) in the stomach of a mature human: (**a**) Pre swallowing tablet. (**b**) Post swallowing tablet. (**c**) After 30 min. (**d**) After 1.5 h. (**e**) After 2.5 h. (**f**) After 3 h.

Figure 6 A Visual Journey Through Stomach and Tablet Interaction. Image a: A baseline photo of the volunteer's stomach before tablet ingestion set the stage by illustrating an empty stomach, priming a comparison for post-tablet ingestion. Image b: Captured after tablet ingestion, this image showcased the tablet within the stomach, providing a clear view of its architectural form and its initial location. Image c: Half an hour after tablet ingestion, the tablet remained within the stomach, albeit altered in its dimensions. This transformation could be attributed to the HPMC content of the tablet, which swelled and hydrated upon contact with stomach fluid.

Image d: Taken 1.5 h post-ingestion, this image highlighted a shifted tablet location. The rapid gastric motility characteristic of fasting states might account for this change. Notably, the altered location was accompanied by a residual stain image of the contrast metal tablet, suggesting adhesion to the gastric mucosa and the split of the tablet into two parts. Image e: At the 2.5-h mark, the tablet's location shifted once again, leaving another stain image in its previous position. This dynamic movement provided further evidence of tablet-mucosa adhesion and its consequential separation. Image f: By the 3-h mark, the tablet had progressed into the small bowel, signifying the final destination of the tablet's journey.

This sequence of images unveiled the tablet’s journey within the stomach over a span of three hours. The tablet's resilience and adhesion to the stomach mucosa were evident as its location shifted, leaving stain images in its wake. Importantly, the study revealed the potential of combining bioadhesion polymer with contrast metal to extend the tablet's residence time in the stomach. This strategy could be particularly effective when the tablet is susceptible to disintegration upon contact with gastric fluid.

Through radiography, the experiment provided insights into the dynamic interaction between bioadhesive tablets and the intricate environment of the human stomach.

**Figure 7:**
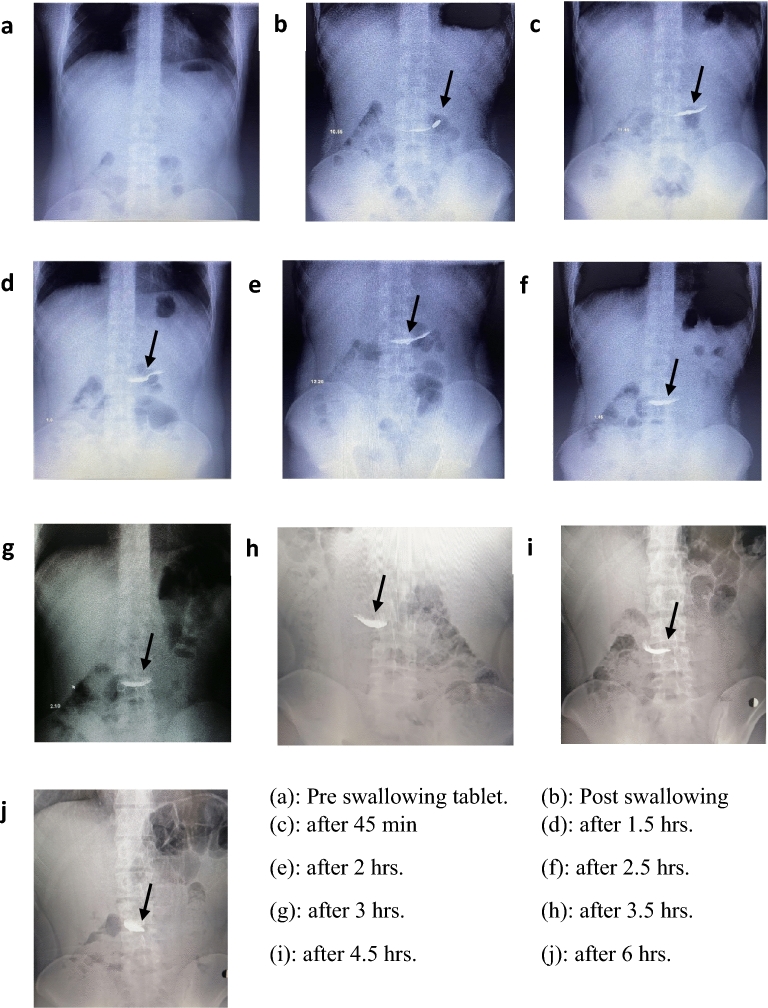
Radiographic images of F6 at different times after oral administration.

Figure 7 illustrates the radiographic behavior of contrast metal tablet (F6) in the stomach of mature humans at different time intervals after administration. Image a: represents the stomach of the volunteer just before ingestion indicating the absence of the contrast metal tablet in the stomach. Image b: shows the tablet in the stomach once ingested indicating the position of the tablet with some stain spreading of the contrast metal although the tablet still has its form. In Image c: is the photo taken at 45 min after ingestion showed the same as before with more spreading of the contrast metal. Image d: is the image taken for the contrast metal tablet after 1.5 h. It indicates the remaining of the contrast metal tablet in the same position in the stomach with widening of the spreading stain to the former position. Image e: shows the same form as image (d) with widening the stain in the forward direction in the stomach after 2 h from ingestion. Image f: indicates the same as image (e) with more symmetrical form of contrast metal stain after 2.5 h. That may indicate the migration of the discharged contrast metal particles to forward continuously and binding of the same particle to another location creating another form. Image g: which is taken after 3 h from the ingestion of the tablet looks completely the same as image (f) indicating the contrast metal particles are still adhering to stomach mucosa. Image h: shows the contrast metal started to move forward led loss the symmetry of the image form after 3.5 h from the tablet taken in the stomach. Image i: showing the contrast metal particles remains in stomach in the same position after 4.5 h of ingestion. Finally, in Image j which is taken after 6 h indicates the reaching of the contrast metal to the small bowel.

**Figure 8:**
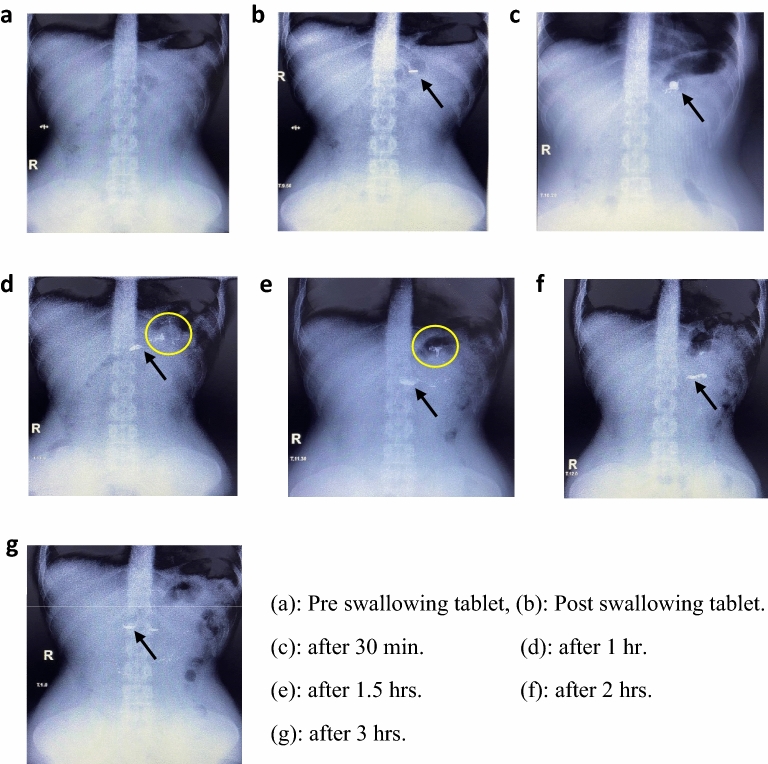
Radiographic images of F7 at different times after oral administration.

Figure 8 represents the radiological images of the contrast metal tablet movement (F7) with the time in the stomach of the mature human. The pre-swallowing image of the empty stomach could be seen from image a. image b shows the tablet in the stomach directly after administration. The contrast metal tablet shows intact form concerning its dimensions. Image c: represents the photo taken after 30 min of ingestion. It shows the tablet located in the stomach with a slight deformation change in the tablet form. Image d: which is taken after 1 h of the contrast metal tablet ingestion shows the migration of the contrast mettle forward leaving a contrast metal stain attached to the previous position. due to the rapid gastric motility in the fasting state and left a stain in the previous position, indicating its adhesion to the gastric mucosa. Images e & f which are taken after 1.5 and 2 h respectively show the same that of image of d with decreasing the lifted contrast metal stain in the tablet previous position. Image g: shows the reaching of the contrast metal particles after 3 h of the ingestion of the tablet to the small bowel.

HPMC E5, a water-soluble polymer, served as the bioadhesive agent for the contrast metal tablet preparation. Upon contact with fluids, this polymer exhibits swelling and dissolution behavior, which likely explains the tablet's deformation shortly after administration. The spreading of the contrast metal image within the stomach can be attributed to the dynamic movements of the stomach.

Although a tablet weighing 1.4 g and adhered to the stomach mucosa on one side might be expected to split due to stomach muscle contractions, the observed splitting did not occur. Instead, the radiographic image revealed the spreading of the contrast metal mass, which gradually increased over time to achieve a symmetrical form.

This behavior can be elucidated as follows: The stomach muscle's contractions instigate continuous movement of the contrast metal bioadhesive tablet. Prior to granulation, the bioadhesive polymer had been thoroughly mixed with the contrast metal using a standard procedure. Since the contrast metal particles are non-compressible and have adhered to the polymer, the tablet's disintegration exposes these particles to the surrounding medium. This situation may lead to the movement of the particles as a cohesive mass, following the direction of the stomach's contraction process.

The combined factors of the tablet’s mass, the stomach muscle contractions, the non-compressibility of the contrast metal, and the adhesion of the tablet's surface to the stomach's lining contribute to the tablet's splitting. Consequently, this process results in leaving behind a contrast metal stain in the original position, while the rest of the tablet migrates forward as a cohesive mass adhered to the stomach's surface, facilitated by the presence of the polymer.

The intricate interplay of these factors provides insights into the tablet's behavior within the stomach, highlighting the complex interaction between bioadhesive agents, contrast metals, and the dynamic environment of the gastrointestinal tract.

## Conclusion

The findings of this study support the notion of utilizing HPMC E5 as a mucoadhesive polymer to enhance the residence time of a dosage form in the stomach. However, it also highlights the potential disruptive impact of using high concentrations of the polymer on the gastric mucosa, which could lead to a form of gastritis. The initial belief was that increasing the polymer concentration would correspondingly increase the residence time, but this study shows that a delicate balance must be maintained to avoid adverse effects on the mucosal layer.

Moreover, the study introduces an innovative approach: the combination of a mucoadhesive polymer with other ingredients, forming a physical mixture that acts as a tool to extend tablet residence time. This was demonstrated through the disintegration behavior of the contrast metal tablet, pressed from granulated barium sulfate mixed with the mucoadhesive polymer. Upon disintegration, the tablet's components reassembled and adhered in a different location within the human stomach. This intriguing observation potentially prolongs the drug's presence in the stomach to over 6 h.

This suggested approach capitalizes on insights gleaned from observing the behavior of contrast metal granules mixed with HPMC E5 within the stomach of a mature human subject. The study underscores the importance of understanding the complex interactions between different components within the gastrointestinal environment, shedding light on new strategies to enhance drug delivery and residence time.

## Data Availability

The datasets generated during the current study are not publicly available because the second part of the research has not yet been finished or published. However, they are available from the corresponding author on reasonable request.

## References

[1] Mudie DM, Amidon GL, Amidon GE (2010). Physiological parameters for oral delivery and in vitro testing. Mol. Pharm..

[2] Sugihara H (2014). Development of a gastric retentive system as a sustained-release formulation of pranlukast hydrate and its subsequent in vivo verification in human studies. Eur. J. Pharm. Sci..

[3] Nayak A, Malakar J, Sen K (2010). Gastroretentive drug delivery technologies: Current approaches and future potential. J. Pharm. Educ. Res..

[4] Kesarla RS, Vora PA, Sridhar BK, Patel G, Omri A (2015). Formulation and evaluation of floating tablet of H2-receptor antagonist. Drug Dev. Ind. Pharm..

[5] Kumar R, Philip A (2007). Gastroretentive dosage forms for prolonging gastric residence time. Int. J. Pharm. Med..

[6] Aoki H, Iwao Y, Mizoguchi M, Noguchi S, Itai S (2015). Clarithromycin highly-loaded gastro-floating fine granules prepared by high-shear melt granulation can enhance the efficacy of *Helicobacter pylori* eradication. Eur. J. Pharm. Biopharm..

[7] Kim J-Y (2014). Efficacy of gastro-retentive forms of ecabet sodium in the treatment of gastric ulcer in rats. Arch. Pharm. Res..

[8] Boddupalli BM, Mohammed ZN, Nath RA, Banji D (2010). Mucoadhesive drug delivery system: An overview. J. Adv. Pharm. Technol. Res..

[9] Chatterjee B, Amalina N, Sengupta P, Mandal UK (2017). Mucoadhesive polymers and their mode of action: A recent update. J. Appl. Pharm. Sci..

[10] Sudhakar Y, Kuotsu K, Bandyopadhyay AK (2006). Buccal bioadhesive drug delivery—A promising option for orally less efficient drugs. J. Control. Release.

[11] Timmins P, Pygall SR, Melia CD (2014). Hydrophilic Matrix Tablets for Oral Controlled Release.

[12] Mašková E (2020). Hypromellose—A traditional pharmaceutical excipient with modern applications in oral and oromucosal drug delivery. J. Control. Release.

[13] Nokhodchi A, Raja S, Patel P, Asare-Addo K (2012). The role of oral controlled release matrix tablets in drug delivery systems. Bioimpacts.

[14] Mukhopadhyay R (2018). Polymers in designing the mucoadhesive films: A comprehensive review. Int. J. Green Pharm. (IJGP).

[15] Punitha S, Girish Y (2010). Polymers in mucoadhesive buccal drug delivery system—a review. Int. J. Res. Pharm. Sci..

[16] Nafee NA, Boraie MA, Ismail FA, Mortada LM (2003). Design and characterization of mucoadhesive buccal patches containing cetylpyridinium chloride. Acta Pharm..

[17] Alanazi FK, Abdel Rahman AA, Mahrous GM, Alsarra IA (2007). Formulation and physicochemical characterisation of buccoadhesive films containing ketorolac. J. Drug Deliv. Sci. Technol..

[18] Mady O (2021). Formulation and bioavailability of novel mucoadhesive buccal films for candesartan cilexetil in rats. Membranes.

[19] Habib F, Abdel Azeem M, Fetih G, Safwat M (2010). Mucoadhesive buccal patches of lornoxicam: I-development and in-vitro characterization. Bull. Pharm. Sci. Assiut.

[20] Singh S, Jain S, Muthu MS, Tiwari S, Tilak R (2008). Preparation and evaluation of buccal bioadhesive films containing clotrimazole. AAPS PharmSciTech.

[21] Nafee NA, Ismail FA, Boraie NA, Mortada LM (2004). Mucoadhesive delivery systems. I. Evaluation of mucoadhesive polymers for buccal tablet formulation. Drug Dev. Ind. Pharm..

[22] Suvarna KS, Layton C, Bancroft JD (2018). Bancroft’s Theory and Practice of Histological Techniques E-Book.

[23] Davies H, Crombie I (2009). What are Confidence Intervals and P-Values?.

[24] Huda W, Abrahams RB (2015). Radiographic techniques, contrast, and noise in x-ray imaging. AJR Am. J. Roentgenol..

[25] Joshi, S. C. & Chen, B. Swelling, Dissolution and disintegration of HPMC in aqueous media. In *13th International Conference on Biomedical Engineering* (eds Lim, C. T. & Goh, J. C. H.) 1244–1247 (Springer, 2009).

[26] Huang X, Gates I (2020). Apparent contact angle around the periphery of a liquid drop on roughened surfaces. Sci. Rep..

[27] Wenzel RN (1936). Resistance of solid surfaces to wetting by water. Ind. Eng. Chem..

[28] Tekade M (2019). Thiolated-chitosan: A novel mucoadhesive polymer for better-targeted drug delivery. Biomaterials and Bionanotechnology.

[29] Zhang Q, Li X, Jasti BR (2021). Role of physicochemical properties of some grades of hydroxypropyl methylcellulose on in vitro mucoadhesion. Int. J. Pharm..

[30] Hassan MA, Barakat NS, El-Badry M, Shehata SM (2011). Formulation and in vitro/in vivo evaluation of naproxen mucoadhesive buccal patches for local effect. J. Drug Deliv. Sci. Technol..

[31] Mortazavi SA, Smart JD (1994). controlled release An in-vitro method for assessing the duration of mucoadhesion. J. Control. Release.

[32] Smart JD, Kellaway IW, Worthington HEC (1984). An in-vitro investigation of mucosa-adhesive materials for use in controlled drug delivery. J. Pharm. Pharmacol..

[33] Smart JD (1991). An m vitro assessment of some mucosa-adhesive dosage forms. Int. J. Pharm..

